# Low- vs. high-dose radiotherapy in Graves’ ophthalmopathy: a retrospective comparison of long-term results

**DOI:** 10.1007/s00066-021-01770-9

**Published:** 2021-04-16

**Authors:** Thomas Weissmann, Sebastian Lettmaier, Anna-Jasmina Donaubauer, Christoph Bert, Manfred Schmidt, Friedrich Kruse, Oliver Ott, Markus Hecht, Rainer Fietkau, Benjamin Frey, Florian Putz

**Affiliations:** 1 Department of Radiation Oncology, Universitätsklinikum Erlangen, Friedrich-Alexander-Universität Erlangen-Nürnberg, Universitaetsstraße 27, 91054 Erlangen, Germany; 2 Translational Radiation Biology, Department of Radiation Oncology, Universitätsklinikum Erlangen, Friedrich-Alexander-Universität Erlangen-Nürnberg, Universitaetsstraße 27, 91054 Erlangen, Germany; 3 Department of Ophthalmology, Universitätsklinikum Erlangen, Friedrich-Alexander-Universität Erlangen-Nürnberg, Schwabachanlage 6, 91054 Erlangen, Germany

**Keywords:** Graves’ ophthalmopathy, Thyroid eye disease, Radiotherapy, Exophthalmos, Low dose radiation therapy

## Abstract

**Purpose:**

Radiotherapy represents an effective treatment option in Graves’ ophthalmopathy (GO), leading to palliation of clinical symptoms. However, there are only a limited number of trials comparing the effectiveness of low- vs. high-dose radiotherapy.

**Methods:**

We analyzed 127 patients treated with radiotherapy for stage 3/4 GO (NOSPECS classification). Patients were treated with single doses of 2.0 Gy (cumulative dose 20 Gy) until 2007, afterwards a single dose of 0.8 Gy (cumulative dose 4.8 Gy) was applied. With a median follow-up-time of 9.0 years, the treatment efficacy (overall improvement, sense of eye pressure, lid edema, ocular motility, exophthalmos, subjective vision, and diplopia) and adverse effects were analyzed by a standardized survey.

**Results:**

Overall, 63.8% described improvement of symptoms after radiotherapy. No significant differences in overall treatment response and improvement of main outcome measures between low- or high-dose radiotherapy treatments are detectable, while low-dose radiotherapy leads significantly more often to retreatment (13.1% vs. 1.7%, *p* = 0.016). The main independent predictor of treatment response is the presence of lid edema (odds ratio, OR, 3.53; *p* = 0.006).

**Conclusion:**

At long-term follow-up, the majority of patients reported palliation of symptoms with limited adverse effects, suggesting clinical effectiveness of radiotherapy for amelioration of GO symptoms independent of low- or high-dose radiotherapy.

## Introduction

Graves’ ophthalmopathy (GO) is the most common cause of proptosis and strabismus in adults [1]. The underlying pathogenic mechanism in GO involves T cells and antibodies directed against antigens associated with membrane receptor proteins expressed in thyroid tissue and orbital fibroblasts, leading to the release of cytokines in the periorbital tissue causing proliferation and expression of immunomodulatory proteins in fibroblasts as well as proliferation of intraorbital adipocytes. The production and deposition of glycosaminoglycans by fibroblasts owing to the hydrophilic property of these macromolecules initially causes enlargement of the extraocular muscles and ultimately leads to fibrosis and functional impairment [2–4].

The pathognomonic set of clinical signs and symptoms significantly reducing patients’ quality of life [5, 6] includes exophthalmos, extraocular muscle dysfunction, diplopia, blurred vision, chemosis, and lid retraction. In severe cases, compressive optic neuropathy may occur, requiring urgent prednisone pulse therapy or orbital decompression [1, 7].

Besides systemic or topical glucocorticoid treatment and surgery, radiotherapy represents one effective treatment option [8, 9]. Clinical trials show equivalent efficacy for radiotherapy (RT) as well as for glucocorticoids with the possibility of a synergistic therapeutic effect for the combined use of both, also leading to reduced side effects overall [10, 11]. Although response rates of approximately 65% are reported following RT in Graves’ disease, the use of RT still remains controversial due to conflicting results from different trials [12, 13].

Varying fractionation schemes for Graves’ disease are used. Gerling et al. were able to show equal benefit for low- and high-dose radiotherapy [14]. The reduced radiation exposure associated with low-dose radiotherapy would be of particular relevance in this disease entity, where concerns of potential future tumor induction weigh more heavily than in life-threatening malignant conditions. The follow-up time of merely 6 months in the study by Gerling et al. does not permit any long-term conclusions, however [14].

Thus, there is still no conclusive evidence on the long-term equivalence of low- and high-dose radiotherapy in patients with Graves’ ophthalmopathy [15].

At the Department of Radiation Oncology of the Universitätsklinikum Erlangen, institutional policy was therefore shifted from high- to low-dose radiotherapy for all patients with Graves’ ophthalmopathy in January of 2007. As long-term follow-up was possible for most patients, the aim of our present work was to perform a detailed long-term comparison of effectiveness and toxicity between low and high-dose radiotherapy in Graves’ ophthalmopathy.

## Experimental section

### Patient population

From December 1984 until October 2018, 252 patients underwent radiotherapy for Graves’ ophthalmopathy at the Department of Radiation Oncology of the Universitätsklinikum Erlangen. 127/252 (50.4%) irradiated patients were eligible for a retrospective survey because they are routinely contacted for telephone interviews, strictly following a standardized questionnaire that had previously been designed for clinical purposes. The retrospective use of patient data is covered by an allowance by the Ethics Committee of the Friedrich-Alexander Universität Erlangen-Nürnberg (ref. 91_20Bc, EORetroRad trial). The study was performed in accordance with the 1964 Declaration of Helsinki and its later amendments. All included patients were diagnosed and assigned by experienced doctors of the ophthalmologic department and further interrogated a second time at the beginning of RT exclusion criteria. Patient suffering from diabetic retinopathy or uncontrolled hypertension were excluded from radiotherapy. From December 1984 until January 2007, all patients (60/127) with Graves’ ophthalmopathy had received a fractionation scheme of 2‑Gy fractions up to 20 Gy, as per institutional treatment policy and consistent with national clinical practice [16]. From January 2007 until October 2018, radiotherapy was changed to a low-dose fractionation scheme of 4.8 Gy in 0.8-Gy single fractions for all patients (61/127). The lower single dose was chosen according to contemporary data suggesting optimal anti-inflammatory effects at single doses of 0.6–0.8 Gy [17]. The cumulative dose of 4.8 Gy was selected so as to minimize the probability of developing a cataract [18]. Patients not benefitting from the procedure would in this case have the lowest chance of suffering long-term side effects due to radiation exposure. In case of re-irradiation, the cumulative dose would still be lower than the traditional cumulative dose of 20 Gy and cumulative doses of 10 Gy have been shown to be equivalent to higher doses [19]. We excluded 4.7% (6/127) of all patients, who received alternative fractionation schemes, from comparative analyses. For the whole cohort, the median follow-up time was 108.3 months (9.0 years; interquartile range 2.9–17.9 years). All patients undergoing a second irradiation series received the same cumulative dose as in the first series. No patient who received 20 Gy of radiation as primary treatment received a second series. The majority (95.3%) of all patients (121/127) had received corticosteroids in addition to radiotherapy, while only 4.7% (6/127) had not. 97.5% (118/121) of these patients received steroids as intravenous infusions before radiotherapy. There was no significant difference in the frequency (96.7% vs. 95.0%, chi-squared *p* = 0.634) or the timing of steroid administration (*p* = 0.385) between the two treatment groups. All patients were classified according to the most commonly applied NOSPECS, EUGOGO, or VISA classification [20]. Table 1 gives a characterization of the analyzed patient cohort.

**Table 1:** Baseline patient characteristics (*n* = 127); RT = Radiotherapy

Baseline patient characteristic	Total cohort (*N* = 127)
*Gender, n (%)*	
Male	39 (30.7%)
Female	88 (69.3%)
*Age at start of first RT—all patients*	
Median (range)	54 (19–85)
*Laterality, n (%)*	
Left eye	22 (17.3%)
Right eye	28 (22.0%)
Both eyes	77 (60.6%)
*Interval from first diagnosis to RT, months*	
Median (range)	4.0 (0.0–95.0)
*Smoker, n (%)*	
Non-smoker	64 (50.4%)
Smoker	63 (49.6%)
*Diabetes, n (%)*	
No diabetes	114 (89.8%)
Diabetes	13 (10.2%)
*Elevated blood pressure, n (%)*	
No hypertension	75 (59.1%)
Hypertension	52 (40.9%)
*Euthyroidism with antithyroid medications, n (%)*	
No euthyroidism achieved	4 (3.1%)
Euthyroidism with antithyroid medications	123 (96.9%)
*Severity of orbitopathy, n (%)*	
NOSPECS class 3	5 (3.9%)
NOSPECS class 4	122 (96.1%)
*Double vision, n (%)*	
No double vision	23 (18.1%)
Double vision present	104 (81.9%)
*Impaired ocular motility, n (%)*	
No impairment of ocular motility	24 (18.9%)
Impairment of ocular motility present	103 (81.1%)
*Exophthalmos, n (%)*	
No exophthalmos	26 (20.5%)
Exophthalmos present	101 (79.5%)
*Impaired vision, n (%)*	
No impaired vision	117 (92.1%)
Impaired vision present	10 (7.9%)
*Sensation of pressure, n (%)*	
No sensation of pressure	75 (59.1%)
Sensation of pressure present	52 (40.9%)
*Eye lid edema, n (%)*	
No eye lid edema	60 (47.2%)
Eye lid edema present	67 (52.8%)
*RT dose and fractionation schedule, n (%)*	
0.8 Gy → 4.8 Gy	61 (48.0%)
2.0 Gy → 20.0 Gy	60 (47.2%)
Other	6 (4.7%)
*Number of RT series, n (%)*	
One RT series	115 (90.6%)
Two RT series	12 (9.4%)
*Corticosteroid treatment, n (%)*	
No corticosteroid treatment	6 (4.7%)
Single course	110 (86.6%)
Multiple courses	11 (8.7%)

*RT* Radiotherapy

### Radiation therapy

**Fig. 1:**
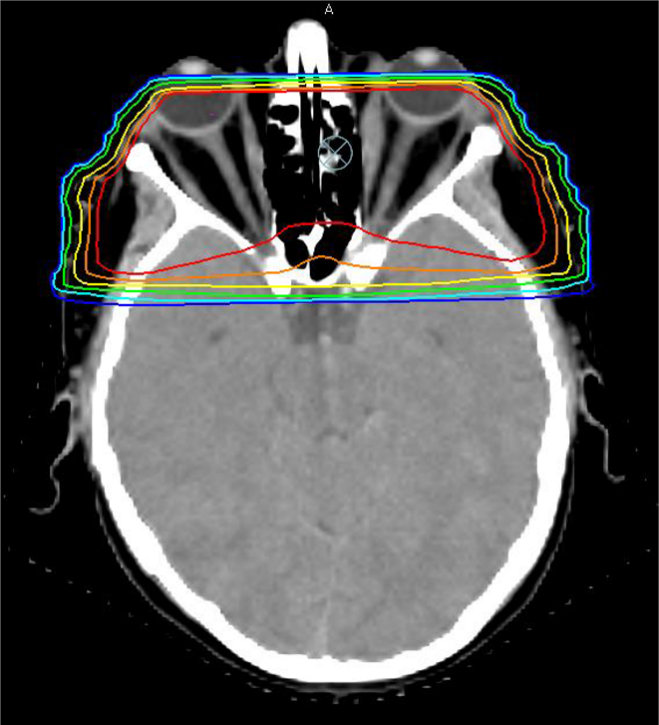
Typical treatment plan in a patient with Graves’ ophthalmopathy. *Orange *planning target volume. Reference point is marked with a *crossed white circle*. Isodoses: *red* 95%, *orange* 90%, *yellow* 80%, *green* 60%, *cyan* 40%, *blue* 30%. Two opposing isocentric beams were used to cover the retrobulbar area. A coincident beam plane just behind the eye lenses was achieved through the adjustment of beam angles

Patients received radiotherapy with a linear accelerator based Oncor™, Primart™ (Siemens Medical Solutions, Erlangen, Germany), or Versa HD™ systems (Elekta AB, Stockholm, Sweden) using 6‑ and 15-MeV photons, respectively. Patients were immobilized in an individually manufactured thermoplastic head mask (Unger, Mülheim-Kärlich, Germany) and treatment planning was performed using the Pinnacle planning system (Philips Radiation Oncology Systems, Fitchburg, WI, USA). Target volumes as well as organs at risk including the eyes, lenses, optic nerves, brain stem, and optic chiasm were delineated on a dedicated planning CT (slice thickness ≤5 mm) as shown in Fig. 1. Target volume was as recommended in the S2e DEGRO (Deutsche Gesellschaft für Radioonkologie e.V.) guideline, i.e., the whole orbit from the tip including the common tendinous ring up to the dorsal two thirds of the orbit. Radiation therapy was applied using two opposing isocentric beams to cover the retrobulbar area. A coincident beam plane just behind the eye lenses was achieved through the adjustment of beam angles. Treatment fields were individually collimated using a multileaf collimator or cast blocks.

### Assessment of treatment efficacy and toxicity

Treatment efficacy was evaluated by reviewing the medical history of each patient, including documentation of the ophthalmologic examination at initial presentation. Initial ophthalmologic examination included the NOSPECS score with examination and evaluation of parameters such as eyelid swelling and conjunctival injection. We also recorded vision and eye motility and the extent of exophthalmos. Assessment of long-term results was based on a retrospective survey in which patients were contacted via telephone to be interviewed strictly following a standardized questionnaire. The contact via phone was chosen to maximize the number of participants available for evaluation. Side effects were recorded for all patients.

### Statistical analysis

From the whole cohort of 127 patients, 48.0% (61/127) received low-dose radiotherapy using a single dose of 0.8 Gy and a total dose of 4.8 Gy, while 47.2% (60/127) received high-dose radiotherapy with single doses of 2 Gy and a total dose of 20 Gy. 4.7% (6/127) received other fractionation schemes and were excluded from comparative analyses.

To test for differences in treatment outcomes between the two fractionation schemes or other categorical variables, a Pearson’s chi-squared test was performed. Differences in continuous variables between patients with and without treatment response were evaluated using the Wilcoxon rank-sum test.

Predictors of therapeutic response were evaluated using univariate and multivariate logistic regression analysis. Presence of lid edema, exophthalmos, diabetes, and hypertension, as well as gender, age, smoking status, and the fractionation scheme were included in the model based on mechanistic and pathophysiologic considerations.

All statistical analyses were performed using IBM SPSS 21 (Armonk, NY, USA). Graphs were generated using Microsoft Excel 2019 (Redmond, WA, USA) and GraphPad Prism 7 (San Diego, CA, USA).

## Results

### Functional outcome and treatment efficacy

In the whole cohort, 63.8% (81/127) described improvement of symptoms following radiotherapy (RT), whereas 36.2% (46/127) reported no improvement. Within the group of patients who responded to the RT, 29.1% (37/127) reported slight improvement, 26.8% (34/127) described marked improvement, and 7.9% (10/127) observed complete resolution of symptoms. Regarding specific symptom categories, 34.6% (44/127) reported improvement of diplopia, 35.4% (45 out of 127 available patients) described improved ocular motility, 50.4% (64/127) observed improvement of exophthalmos, 7.1% (9 out of 127 available patients) reported improved subjective vision, and 38.8% (33 out of 85 available patients) had improvement in sense of pressure (Table 2). Only 2.4% (3/127) reported worsening of symptoms after radiotherapy. The majority of the patients, 82.7% (105/127), reported that they would undergo treatment again if needed and 62.2% (79/127) of patients received no further surgery. All patients undergoing surgery underwent surgery due to restorative reasons. No patient underwent surgery due to acute compressive symptoms. At least 9.4% (12/127) of patients went on to receive a second series of radiation therapy at a later stage (Table 2).

**Table 2:** Functional outcome and comparison between fractionation schemes

Functional outcome	Total cohort (*N* = 127)	0.8 Gy ⇨ 4.8 Gy (*N* = 61)	2.0 Gy ⇨ 20 Gy (*N* = 60)
*Improvement of symptoms, n (%)*	*p* *=* *0.615 (chi-squared)*		
No improvement	46 (36.2%)	23 (37.7%)	20 (33.3%)
Improvement after RT	81 (63.8%)	38 (62.3%)	40 (66.7%)
Slight improvement	37 (29.1%)	17 (27.9%)	20 (33.3%)
Marked improvement	34 (26.8%)	16 (26.2%)	16 (26.7%)
Complete response of symptoms	10 (7.9%)	5 (8.2%)	4 (6.7%)
*Improvement of diplopia, n (%)*	*p* *=* *0.159 (chi-squared)*		
No improvement	83 (65.4%)	44 (72.1%)	36 (60.0%)
Improvement after RT	44 (34.6%)	17 (27.9%)	24 (40.0%)
*Improvement of ocular motility, n (%)*	*p* *=* *0.332 (chi-squared)*		
No improvement	80 (64.0%)	42 (68.9%)	35 (60.3%)
Improvement after RT	45 (36.0%)	19 (31.1%)	23 (39.7%)
*Improvement of exophthalmos, n (%)*	*p* *=* *0.236 (chi-squared)*		
No improvement	63 (49.6%)	33 (54.1%)	26 (43.3%)
Improvement after RT	64 (50.4%)	28 (45.9%)	34 (56.7%)
*Improvement of subjective vision, n (%)*	*p* *=* *0.435 (chi-squared)*		
No improvement	117 (92.9%)	58 (95.1%)	54 (91.5%)
Improvement after RT	9 (7.1%)	3 (4.9%)	5 (8.5%)
*Improvement in sense of pressure, n (%)*	*p* *=* *0.369 (chi-squared)*		
No improvement	52 (61.2%)	22 (66.7%)	29 (56.9%)
Improvement after RT	33 (38.8%)	11 (33.3%)	22 (43.1%)
*Worsening of symptoms, n (%)*	*p* *=* *0.549 (chi-squared)*		
No worsening	124 (97.6%)	60 (98.4%)	58 (96.7%)
Worsening after RT	3 (2.4%)	1 (1.6%)	2 (3.3%)
*Would undergo treatment again, n (%)*	*p* *=* *0.085 (chi-squared)*		
Would not undergo treatment again	22 (17.3%)	7 (11.5%)	14 (23.3%)
Would undergo treatment again	105 (82.7%)	54 (88.5%)	46 (76.7%)
*Surgery after RT, n (%)*	*p* *=* *0.072 (chi-squared)*		
No surgery	79 (62.2%)	33 (54.1%)	42 (70.0%)
Surgery needed	48 (37.8%)	28 (45.9%)	18 (30.0%)
Second series performed*, n (%)	*p* *=* 0.016* (chi-squared)		
No second series performed	115 (90.6%)	53 (86.9%)	59 (98.3%)
Second series performed	12 (9.4%)	8 (13.1%)	1 (1.7%)

Functional outcome and comparison between fractionation schemes section is divided by subheadings. This should provide a concise and precise description of the experimental results and their interpretation as well as the experimental conclusions that can be drawn. The comparison of 6 × 0.8 (Σ = 4.8 Gy) against 10 × 2.0 Gy (Σ = 20 Gy) was analyzed using a chi-squared test, the *p*-value is depicted in the table *RT* radiotherapy

*significant association (*p* < 0.05)

**Fig. 2:**
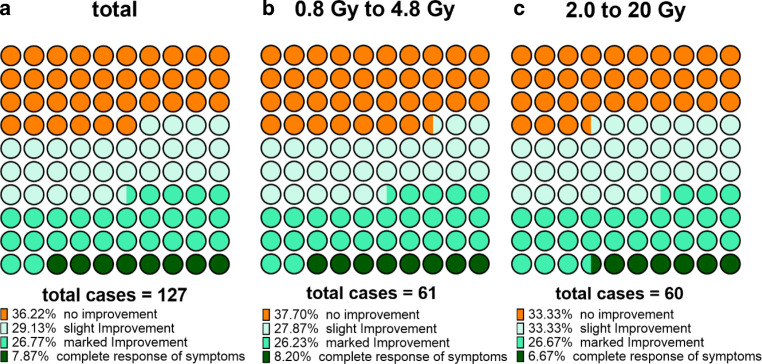
Parts of whole chart of response rate. The single charts represent the number of patients who showed a response to treatment or failed to respond. The charts show the response rate of all patients (**a**) and for patients treated with low-dose (**b**) and high-dose radiotherapy (**c**) separately

When comparing the low-dose (6 × 0.8 Gy, *n* = 61) and the high-dose (10 × 2.0 Gy, *n* = 60) fractionation schemes, no significant difference in overall improvement of symptoms was found (improvement reported in 62.3% vs. 66.7% of patients; *p* = 0.615; Fig. 2). In addition, regarding specific symptoms, no significant difference was found between low- and high-dose radiotherapy (Table 2). Interestingly, within the low-dose cohort, 88.5% of patients reported that they would undergo treatment again compared to 76.7% of those treated with high-dose RT (*p* = 0.085). Subsequent surgery was more often performed in patients treated with low-dose RT (45.9% vs. 30.0%; *p* = 0.072), however. Patients treated with low-dose radiation went on to receive a second series of radiotherapy significantly more frequently than patients treated with high-dose RT (13.1% vs. 1.7%; *p* = 0.016; Table 2).

### Adverse effects

In the whole cohort, only 8.7% (11/127) reported any adverse effects. Transient dry eyes and conjunctivitis were the most frequently reported adverse effects (3.9%; 5/127). Other low-grade side effects were very rare (Table 2). One patient (0.8%) reported the occurrence of a cataract that could be attributed to radiation. We also observed no case of documented retinopathy attributed to radiotherapy. No other high-grade or chronic toxicity was observed and, importantly, no case of secondary malignancy could be identified within the whole follow-up period. Notably, 21.3% (27/127) of the included patients showed a follow-up of more than 20 years with no signs of cranial malignancies.

When comparing low-dose and high-dose radiotherapy, adverse effects were more frequently reported in the low-dose cohort (14.8% vs. 3.3%; *p* = 0.029), which was mainly accounted for by a more frequent reporting of dry eyes/conjunctivitis in the low-dose group (8.2% vs. 0%; *p* = 0.024). The only case of radiotherapy-related cataract was observed in the high-dose group (Table 3). When further analyzing the unexpectedly high rate of adverse effects in the low-dose group, we found that patients who had received a second series of radiation were overrepresented in the group reporting adverse events. 44% (4/9) of the patients experiencing adverse effects in the low-dose group had received a second series of radiation, while only 7.7% (4/52) of patients without adverse events were treated with a second radiotherapy series (*p* = 0.003, chi-squared). Similarly, in the whole cohort of 127 patients, the risk of adverse effects was significantly increased in the subgroup that had received a second series of radiation (relative frequency of adverse events 33.3% vs. 6.1%, chi-squared *p* < 0.001).

**Table 3:** Toxicity and comparison between fractionation schemes

Toxicity	Total cohort (*N* = 127)	0.8 Gy ⇨ 4.8 Gy (*N* = 61)	2.0 Gy ⇨ 20 Gy (*N* = 60)
Any adverse effects*, *n* (%)	*p* = 0.029* (chi-squared)		
No adverse effects	116 (91.3%)	52 (85.2%)	58 (96.7%)
Any adverse effects	11 (8.7%)	9 (14.8%)	2 (3.3%)
Dry eyes/conjunctivitis*, *n* (%)	*p* = 0.024* (chi-squared)		
No dry eyes/conjunctivitis	122 (96.1%)	56 (91.8%)	60 (100.0%)
Dry eyes/conjunctivitis after RT	5 (3.9%)	5 (8.2%)	0 (0.0%)
Impairment of taste or smell, *n* (%)	*p* = 0.991 (chi-squared)		
No impairment of taste or smell	125 (98.4%)	60 (98.4%)	59 (98.3%)
Impairment of taste or smell after RT	2 (1.6%)	1 (1.6%)	1 (1.7%)
Headache, *n* (%)	*p* = 0.319 (chi-squared)		
No headache	126 (99.2%)	60 (98.4%)	60 (100.0%)
Headache after RT	1 (0.8%)	1 (1.6%)	0 (0.0%)
Impaired vision, *n* (%)	*p* = 0.319 (chi-squared)		
No Impaired vision	126 (99.2%)	60 (98.4%)	60 (100.0%)
Impaired vision after RT	1 (0.8%)	1 (1.6%)	0 (0.0%)
Eye lid edema, *n* (%)	*p* = 0.319 (chi-squared)		
No eye lid edema	126 (99.2%)	60 (98.4%)	60 (100.0%)
Eye lid edema after RT	1 (0.8%)	1 (1.6%)	0 (0.0%)
Cataract, *n* (%)	*p* = 0.311 (chi-squared)		
No cataract	126 (99.2%)	61 (100.0%)	59 (98.3%)
Cataract after RT	1 (0.8%)	0 (0.0%)	1 (1.7%)

The comparison of 6 × 0.8 (Σ = 4.8 Gy) against 10 × 2.0 Gy (Σ = 20 Gy) was analyzed using a chi-squared test, the *p*-value is depicted in the table

*RT* radiotherapy

*significant association (*p* < 0.05)

### Determinants of therapeutic response

To evaluate predictors of therapeutic response including the use of low- vs. high-dose radiotherapy, we performed univariate and multivariate logistic regression analysis (Table 4).

**Table 4:** Multivariate logistic regression analysis of predictors of therapeutic response in patients receiving 6 × 0.8 (Σ = 4.8 Gy) or 10 × 2.0 Gy (Σ = 20 Gy; *N* = 121)

	Univariate		Multivariate	
Parameter	Odds ratio for therapeutic response	Univariate* p*-value	Odds ratio for therapeutic response	Multivariate* p*-value
Presence of lid edema, yes vs. no*	3.15	0.004	3.53	0.006*
Gender, female vs. male*	3.43	0.003	3.27	0.012*
Diabetes vs. no diabetes	0.51	0.276	0.28	0.096
Age, ≥50 vs. <50 years	0.90	0.784	1.53	0.404
Hypertension vs. none	0.82	0.613	1.41	0.474
Presence of exophthalmos, yes vs. no	1.72	0.242	1.78	0.301
Smoker vs. non-smoker	0.71	0.365	0.80	0.602
Diplopia or restriction of eye movement, yes vs. no	0.51	0.223	0.44	0.210
Fractionation, 6 × 0.8 vs. 10 × 2 Gy	0.83	0.616	0.75	0.538

*significant association (*p* < 0.05)

**Fig. 3:**
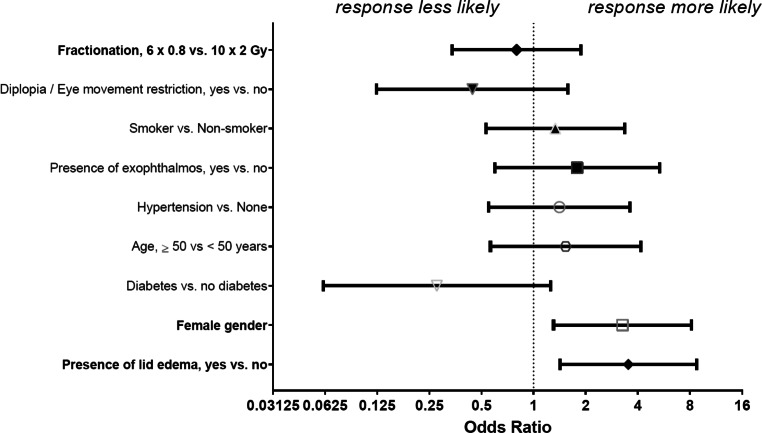
Forest plot of the multivariate logistic regression analysis illustrating independent factors associated with therapeutic response in radiotherapy for Graves’ ophthalmopathy. Calculated odds ratios with 95% confidence intervals are shown. Note: female gender and the presence of lid edema were the only factors significantly associated with therapeutic response, whereas low- vs. high-dose radiation was not (*bold*)

Presence of lid edema, exophthalmos, diabetes, or hypertension, as well as gender, age, smoking status, indicators of muscle involvement, and the fractionation scheme were included in the model based on mechanistic and pathophysiologic considerations. In univariate and multivariate logistic regression analysis, only the initial presence of eyelid edema (OR for therapeutic response 3.53; *p* = 0.006) and female gender (OR 3.27; *p* = 0.012) were positive predictors of therapeutic response. Fractionation scheme (high- vs. low-dose) was not a significant determinant, neither in univariate (*p* = 0.616) nor in multivariate analysis (*p* = 0.538; Fig. 3).

## Discussion

GO is known to pass through different stages. The first stage of progressive deterioration extends to a timespan of about 18–36 months, followed by a brief plateau phase and a protracted phase of incomplete recovery. The disease ends in a burnout phase in which the disease becomes static [20]. The self-limiting course with a tendency towards spontaneous remission limits the validity of conclusions drawn from retrospective studies and even from prospective double-blind controlled trials investigating the outcome of therapy in GO. This may also be part of the reason why studies investigating the effect of radiotherapy on GO show variable results. For example, in their study, Gorman et al. [12] showed no or very little improvement in patients with mild to moderate ophthalmopathy, whereas Beckendorf et al. reported a partial response in 50% of the patients and even a good or excellent response in another 26% of the patients. The inclusion of patients suffering from GO for up to several years has to be regarded as a limitation in Gorman’s study design [12, 21]. Furthermore, the sham irradiation they carried out has to be understood as a pseudo-sham irradiation, as the untreated eye still received a dose of 0.4 Gy, a dose well known to cause immunological and especially anti-inflammatory effects [22, 23]. Mouritis and colleagues on the other hand carried out a study comparing radiotherapy with a cumulative dose of 20 Gy to sham irradiation, which showed a symptomatic improvement especially related to eye motility, with 25% of the patients in the RT group being spared the need for secondary corrective surgery for strabismus. Furthermore, an accelerated decrease in the clinical disease activity score was observed in the RT group [10, 13, 24].

Regarding single and cumulative doses used in RT of GO there are several different dose schedules in current use. Historically, several studies have applied single doses ranging from 0.5 up to 2.0 Gy and cumulative doses of 16 to 20 Gy. The need for higher single and cumulative doses needs to be questioned [19, 25, 26]. Data on the use of single fractions lower than 1 Gy are very scarce.

Gerling et al. investigated the effect of dose schedules employing eight fractions of 0.3 Gy vs. eight fractions of 2 Gy in 86 patients [14]. Outcome measures included clinical appearance of the eye region, exophthalmos, range of vertical eye motility, eye muscle thickness, and individual patient complaints. No significant differences were observed between the two groups [14]. Gerling’s assumption that the effective total dose is unlikely to fall within the range from 2.4 to 20 Gy may be challenged considering that it is well known that the production of glycosaminoglycans by fibroblasts decreases already at a cumulative dose of less than 10 Gy. Other shortcomings of this study include the evaluation of patients independently of their disease stage and the short follow-up, possibly missing instances of delayed recurrence or secondary worsening that—as previously mentioned—are known to commonly occur in the course of Graves’ disease. The short follow-up further limits the possibility of investigating delayed improvement since improvements in soft tissue swelling, ocular motility, and visual acuity are reported up to 52 weeks after treatment and this tendency for delayed improvement may well continue for even longer follow-up periods [27].

Choosing an initial course of low-dose RT, which may itself be sufficient for sustained disease control in most patients, has been suggested to individually tailor the administered total dose to a patient’s needs by leaving the option for a second “salvage” course of radiotherapy in case of an insufficient response to the initial course. So, in a second series of RT, the cumulative dose can be topped up to a total of 10 Gy which has shown equivalent results to even higher doses in earlier studies [19]. In our present series, only 13.1% (8/61) of patients received an additional series of radiotherapy following low-dose RT, mainly due to therapy-refractory symptoms and high disease burden. While this suggests that one series of low-dose RT is sufficient in most patients, due to limitations imposed by design and treatment selection in the present series, no conclusion about the effectiveness of a second salvage series after failure of a first series of low-dose RT is possible based on the present analysis. Importantly, however, in the present cohort the risk for adverse effects was profoundly increased in patients receiving a second series of radiation therapy. Therefore, based on our analysis, the routine prescription of a second salvage RT series in case of insufficient treatment response to an initial series of low-dose RT cannot be recommended at this stage. Instead, in view of a significantly increased risk of side effects, albeit low grade, a second series of radiation should be reserved for selected cases only.

A limitation of the present study has to be seen in the timepoint of data acquisition. With all patients being interrogated during a single telephone consultation, our results are clearly subjectively biased due to the different time spans from radiotherapy to consultation. Nevertheless, due to a high level of physiologic and psychologic suffering, we are convinced that the reported data reflect patients’ symptom burden correctly. An important additional consideration for the interpretation of the study results are differences in the follow-up period, which could have influenced results. However, with follow-up periods of multiple years for both study groups, patients can be expected to be in a stable phase of their disease. In addition, the longer follow-up period of patients in the high-dose group would be expected to result in better remission than in patients with a shorter follow-up time in the low-dose group, which supports the conclusion that low-dose radiotherapy was in fact not inferior. Another drawback has to be seen in the simplified acquisition of patient symptoms. Although several different questionnaires like the clinical activity score (CAS) are available, we chose to simplify our interrogation. Due to the retrospective character of the interrogation, extensive collection of data is not possible and even confusing, as patients do not have the understanding for a vast number of symptoms. We distinctively chose symptoms that are easy to describe, and we think burden the patients most.

No secondary malignancies were observed in the present series, even in the group of patients who had been followed for more than 20 years. The risk of induction of fatal malignancies is calculated at 0.6%, while the risk of tumor induction in general is estimated at 1.2%, generally limiting the use of RT as a treatment option for benign conditions to patients older than 30 years [28]. With cumulative dose playing a significant role in the potential induction of secondary malignancies, the reduction of cumulative dose was the most distinctive feature of our study, allowing minimization of the risk of secondary malignancies without compromising the therapeutic effect. Although as warned by the ICRP (International Commission of Radiation Protection), the concept of effective dose should not be used for calculation of cancer risk in specific irradiated populations such as patients undergoing radiotherapy [29], it is assumed that a reduction in cumulative dose could potentially lower the lifetime attributable risk for cancer [30].

Although side effects such as retinopathy and chronic xerophthalmia are reported in the literature, no such cases could be confirmed in our cohort [31] Two patients reported a significant reduction in taste and smell, while one patient complained of impaired vision following radiotherapy. Review of the patients’ radiotherapy treatment plan, however, did not reveal any hotspots in the oral cavity, the olfactory region, or the retina to explain this. One patient developed a cataract following therapy at an early stage, which may potentially be linked to radiotherapy. In summary radiotherapy was well tolerated in the present long-term assessment, with 82.7% of the patients reporting to be willing to repeat the procedure if necessary.

Several studies use criteria such as eyelid swelling, eye muscle motility, etc. as a measure for treatment success. RT has been shown to lead to a significant improvement as judged by these criteria without these really giving a good reflection of the patient’s subjective sense of improvement. In our patient cohort, 63.8% (81/127) of the patients reported a benefit in terms of symptoms following RT, with 26.8% (34/127) reporting profound improvement and 7.9% (10/127) even reporting complete reduction of symptoms following RT. No significant differences were shown between fractionation schemes in our study, making a strong case for low-dose radiation therapy in GO.

Of the patients in our cohort, 37.8% (48/127) required further treatment in the form of surgery to deal with treatment-refractory double vision. RT in these cases may still hold a value, diminishing inflammation as a fundamental requirement for the ensuing surgical intervention [32, 33]. Comparing both radiation schemes, a slight tendency towards more frequent interventions following lower RT doses has been observed. This may potentially be attributed to differences in biological effects or, alternatively, may be an effect of the ongoing optimization in surgical techniques. In summary, there was no significant benefit for the application of higher doses seen in the present study. In view of the mathematical risk of inducing malignancies, there is no justification for the continued application of the higher doses still commonly employed in the clinical routine for treatment of GO.
